# Photodegradation Mechanisms and Anti-Aging Strategies of Wood Coatings: A Comprehensive Review

**DOI:** 10.3390/polym18091090

**Published:** 2026-04-29

**Authors:** Meng Xia, Hanyun Gao, Xinhao Feng, Xinyou Liu

**Affiliations:** 1 College of Furnishing and Industrial Design, Nanjing Forestry University, Nanjing 210037, China; xiameng@njfu.edu.cn (M.X.); ghy0128@njfu.edu.cn (H.G.); fengxinhao@hotmail.com (X.F.); 2 Co-Innovation Center of Efficient Processing and Utilization of Forest Resources, Nanjing Forestry University, Nanjing 210037, China

**Keywords:** antioxidants, HALS, nanocomposite coatings, photodegradation, UV absorbers, wood coatings

## Abstract

Wood coatings play a critical role in protecting wood substrates from environmental degradation, particularly ultraviolet (UV)-induced photodegradation. This review comprehensively examines the mechanisms of wood coating photodegradation, the factors influencing their durability, and current anti-aging strategies. Photodegradation arises from polymer chain scission, chemical structure reorganization, and photo-oxidation of lignin and cellulose, leading to coating chalking, cracking, gloss loss, and color changes, ultimately compromising wood mechanical properties and service life. Key anti-aging strategies include UV absorbers, which convert harmful UV radiation into heat; hindered amine light stabilizers (HALSs) that capture free radicals and quench excited-state molecules; barrier and shielding materials that form dense physical or nanostructured networks to block UV penetration and enhance mechanical and water resistance; and antioxidants that neutralize free radicals or decompose peroxides at the molecular level. Each approach can be employed individually or synergistically to enhance coating durability. Challenges remain in achieving long-term outdoor stability, balancing transparency and UV shielding, optimizing nanoparticle dispersion, and maintaining the activity of natural antioxidants. Future research should focus on multifunctional composite coatings integrating bio-based materials and nanotechnology, smart responsive systems, adaptive protection mechanisms, and standardized long-term evaluation protocols. These advancements will facilitate the development of high-performance, sustainable wood coatings and promote the value-added utilization of wood resources.

## 1. Introduction

Wood is widely utilized in construction, furniture, and handicraft applications due to its excellent strength-to-weight ratio, unique aesthetic texture, and favorable processability [1]. Wood coatings serve as a primary means for surface functionalization, not only enhancing the natural beauty of wood but also providing indispensable protection against environmental degradation, thereby extending the service life of wood products [2]. However, when coated wood is exposed to outdoor conditions, sunlight—particularly its ultraviolet (UV) component—triggers complex photochemical aging reactions, which significantly limit the durability of wood products [3,4].

Photodegradation of wood coatings typically manifests as cracking, chalking, loss of gloss, and color changes. At the molecular level, these phenomena arise from polymer chain scission and chemical structure rearrangement within the coating matrix [5]. Ultraviolet radiation can penetrate the coating and interact with the wood substrate, inducing lignin photodegradation and cellulose structural damage [3,6]. The conjugated double bonds and phenolic hydroxyl groups within lignin are highly photoactive; upon UV absorption, they readily initiate radical chain reactions, leading to polymer chain breakage and chromophore formation [3,7]. This not only causes yellowing and darkening of the wood but also compromises its mechanical properties and structural integrity [6], severely affecting the preservation and utility of outdoor wooden furniture, architectural facades, and cultural heritage objects.

Photodegradation poses dual challenges for the use and preservation of wood. In outdoor applications, coating failure accelerates substrate weathering, increasing maintenance costs and shortening service life [4]. For museum-held wooden artifacts, UV-induced irreversible discoloration and surface deterioration directly damage their artistic value and historical information [8]. Conventional protection strategies primarily rely on inorganic nanosized metal oxides or organic UV absorbers [9,10]; however, the former often suffer from poor dispersion and weak interfacial adhesion, while the latter may face limited durability and potential environmental risks [10].

In recent years, extensive research has focused on enhancing the photostability of wood coatings. Mechanistic studies have systematically elucidated the photodegradation pathways of wood components, identifying lignin and extractives as major contributors to discoloration [3,6], and have revealed how external factors such as light intensity, temperature, and humidity modulate aging processes [4]. In terms of protective technologies, research has shifted from single-function coatings toward multifunctional synergistic systems, encompassing surface coating modification, chemical treatment, and bio-based anti-photoaging films [9].

**Figure 1:**
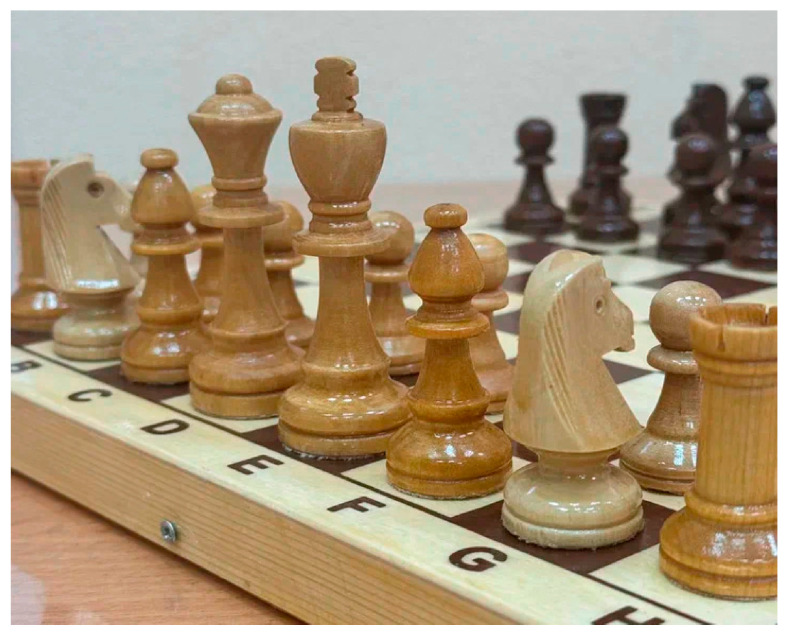
Wooden chess pieces coated with FDCA-based alkyd resins (ARs) [13].

Notably, bio-based additives and organic–inorganic hybrid strategies have emerged as research hotspots. For example, composites of cellulose nanocrystals and CeO_2_ nanoparticles can significantly enhance UV shielding performance [11]; chitosan–ZnO nanocomposite coatings effectively improve wood photostability [12]; and lignin and its derivatives, owing to their aromatic ring structures and UV absorption properties, provide both UV shielding and functionalization of coatings [13]. Beyond compositional advantages, the practical appearance and protective quality of bio-based coatings are equally important for wood finishing. Figure 1 shows a set of wooden chess pieces coated with FDCA-based alkyd resins (ARs), a bio-derived coating system. The image demonstrates excellent leveling, high gloss retention, and clear preservation of wood grain, while providing effective moisture and dust resistance. Such visual examples highlight that sustainable coatings can simultaneously achieve aesthetic appeal and robust protection, bridging the gap between material development and practical application [14]. Moreover, superhydrophobic modifications and synergistic use of photostabilizers offer novel approaches to further improve coating durability [15,16].

**Figure 2:**
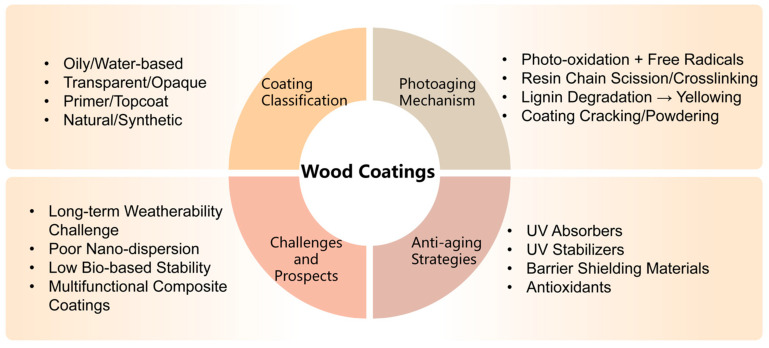
Overview framework of wood coating photodegradation and anti-aging strategies.

The photodegradation of wood coatings involves complex interfacial chemistry and environmental response mechanisms. Enhancing their anti-aging performance requires a multi-dimensional approach, including material design, structural optimization, and functional synergy. While previous studies and reviews have addressed photodegradation mechanisms or specific stabilization methods, they often focus on isolated aspects and lack a systematic integration of mechanisms with anti-photoaging strategies. This review establishes a multi-scale framework linking coating mechanisms, stabilization approaches, and application performance. It systematically compares four major strategies—UV absorbers, HALS, barrier/shielding materials, and antioxidants—and highlights emerging trends such as bio-based additives and multifunctional nanocomposites, providing clear guidance for the development of durable, high-performance wood coatings. This review systematically summarizes the characteristics and mechanisms of wood coating photodegradation, highlights recent advances in anti-photoaging modifications, and analyzes the advantages and limitations of current technologies, aiming to provide theoretical guidance and practical insights for the development of high-performance wood coatings and the value-added utilization of wood resources (Figure 2). A brief methodology describing the purpose, scope, and analytical framework of this review is provided in Section 2.

## 2. Methodology of the Review

This review is conducted as a narrative and integrative literature review, aiming to systematically synthesize current knowledge on the photodegradation mechanisms and anti-photoaging strategies of wood coatings.

Purpose:

The primary purpose of this review is to bridge the gap between fundamental photodegradation mechanisms and practical stabilization strategies, providing a structured understanding of how different approaches contribute to coating durability.

Scope:

The scope of this review focuses on wood coating systems, including solvent-based, waterborne, and UV-curable coatings, with particular emphasis on UV-induced degradation processes and anti-photoaging modifications. The literature considered mainly includes peer-reviewed journal articles related to polymer degradation, wood science, and coating technologies.

Function:

This review serves both a descriptive and analytical function, summarizing existing research while comparatively evaluating different stabilization strategies in terms of mechanism, effectiveness, and applicability.

Type of analysis and intent:

An integrative and comparative analysis is adopted. The review classifies and discusses four major strategies—UV absorbers, HALS, barrier/shielding materials, and antioxidants—and further examines their synergistic effects and limitations. The intent is to provide theoretical guidance and practical insights for the design of high-performance and sustainable wood coatings.

## 3. Classification and Aging Mechanisms of Wood Coatings

Wood coatings serve as critical protective materials for wood, preventing direct exposure of the wood surface to environmental factors such as air, moisture, light, and microbial attack. This protective function effectively slows down dimensional changes due to water absorption, shrinkage-induced cracking, discoloration, and biological degradation. By forming a continuous layer on the wood surface, coatings not only provide a physical barrier that limits the penetration of water and oxygen but also absorb or reflect ultraviolet (UV) radiation, thereby reducing the direct photochemical damage to the wood substrate. Additionally, the properties of the resin matrix, pigments, and additives collectively determine key performance metrics such as adhesion, flexibility, and weather resistance, which in turn affect the overall durability of the coating system.

### 3.1. Classification and Protective Mechanisms of Wood Coatings

**Figure 3:**
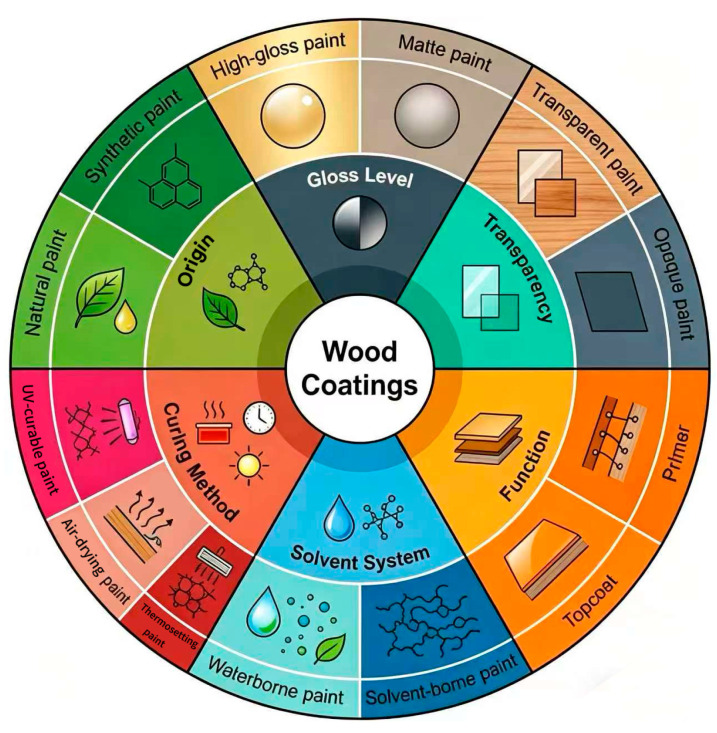
Classification of wood coatings.

Wood coatings can be categorized based on multiple criteria, including gloss, transparency, function, solvent type, curing method, and source. These classifications reflect differences in film formation, interfacial interactions, and environmental response mechanisms, which directly influence the protective pathways and anti-aging performance of the coatings (Figure 3).

- Gloss: Coatings can be high-gloss or matte. High-gloss coatings exhibit a dense, continuous film with strong surface reflection and vivid color, whereas matte coatings provide a softer finish with some light diffusion.
- Transparency: Coatings are either transparent, preserving the natural texture and pattern of the wood for decorative purposes, or opaque, providing strong coverage for discolored or uneven substrates.
- Function: Primers primarily enhance adhesion between the substrate and the topcoat while offering sealing protection. Topcoats serve both decorative and protective roles, imparting aesthetics and durability.
- Solvent Type: Solvent-based coatings use organic solvents, forming dense films with good wetting properties but higher volatile organic compound (VOC) emissions. Water-based coatings use water as the primary dispersing medium, offering improved environmental performance, aligning with current green coating trends.
- Curing Method: Thermosetting coatings require heat to achieve crosslinking, forming a stable three-dimensional network, while air-drying coatings cure at ambient temperature via solvent evaporation or oxidative polymerization, facilitating easier application.
- Source: Natural coatings, such as traditional lacquer, are derived from plant exudates, featuring unique decorative properties but requiring strict drying conditions and longer curing times. Synthetic coatings are manufactured from polymers, offering tunable properties and widespread application in modern wood finishing.

The protective effects of wood coatings can be analyzed at macro, micro, and molecular scales:

- Macro-scale: The coating forms a continuous, dense film on the wood surface, preventing water, air, and contaminant ingress while reflecting part of the UV radiation, thereby delaying photodegradation [17,18].
- Micro-scale: The crosslinked network and micropores of the coating restrict the diffusion of water and oxygen, while interfacial adhesion between the coating and cellulose limits moisture transport along fibers, reducing wood swelling and shrinkage [19].
- Molecular-scale: Interactions between resin molecules and wood chemical components create a highly crosslinked, dense network. UV absorbers and radical scavengers neutralize free radicals generated by photochemical reactions, while hydrogen bonding or van der Waals interactions between resin molecules and cellulose hydroxyl groups enhance interfacial stability, preventing hydrolysis and lignin degradation [20].

### 3.2. Factors Affecting Photodegradation of Wood Coatings

The photodegradation performance of wood coatings is influenced by material properties, environmental conditions, wood treatment processes, and additives, representing a synergistic effect of multiple factors [21].

Material and additive effects: The resin type and film-forming temperature directly affect light resistance and mechanical adaptability. Low-temperature latex forms softer films that accommodate micro-deformations, enhancing aging resistance [22]. Pure acrylic resins offer good anti-yellowing performance but lower hardness, requiring increased crosslink density for improvement [23]. The synergistic addition of UV absorbers and hindered amine light stabilizers (HALS) is crucial for delaying aging; for example, introducing nanosized TiO_2_ and UV531 in industrial coatings significantly improves yellowing resistance [24]. Nanomaterials such as TiO_2_ and lignin nanospheres enhance UV shielding, with 1% lignin nanospheres reducing UVA transmittance to below 10%. Chemical treatments, such as furfuryl alcohol modification, can reduce free radical generation at the substrate level, improving wood durability.

Environmental factors: UV irradiation duration and wavelength determine aging rate, with short-wavelength UV (e.g., UVA-340) causing higher energy exposure and faster gloss loss [25]. Extended exposure further reduces surface gloss and increases cracking. Temperature and humidity cycles induce swelling and shrinkage, generating internal stresses that lead to cracking, while elevated temperature accelerates free radical reactions. Acid rain and particulate pollutants alter wood surface chemistry and increase chromophore formation, accelerating photodegradation.

Wood treatment and synergistic optimization: Surface coating improves UV resistance but may develop cracks over time; chemical modifications, such as isopropyl glycidyl ether treatment, extend service life. Waxing with solvent-based coatings offers more uniform coverage and better adhesion than traditional hot waxing. Current trends focus on multi-factorial optimization, using nanomaterials, light stabilizers, and chemical modifications in combination to increase photostability by over 50%. Advanced approaches, such as core–shell UV-shielding materials and UV-LED curing with real-time colorimetric analysis, enable precise evaluation and improvement of wood coating photostability.

### 3.3. Photodegradation Mechanisms of Wood Coatings

**Figure 4:**
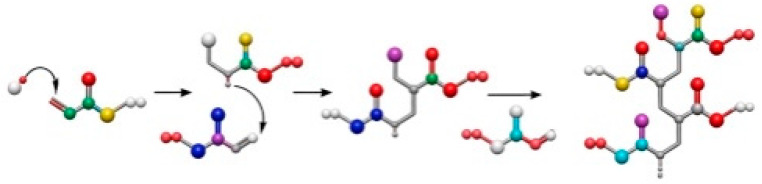
Reaction of radical polymerization of acrylate groups [28].

The photodegradation of wood coatings is dominated by oxidative reactions in the resin [26]. When exposed to UV light (290–400 nm), active functional groups in the resin absorb photon energy, generating free radicals and initiating chain oxidative reactions, including backbone scission, crosslinking, and functional group degradation. Free radicals rapidly react with oxygen to form peroxyl radicals (ROO•), which attack neighboring chains, accelerating molecular weight changes, leading to surface embrittlement, chalking, and reduced barrier properties against moisture and oxygen, weakening wood protection [27]. Furthermore, photodegradation induces measurable chemical modifications in both coating and wood substrate, as evidenced by FTIR spectroscopy. For example, studies have reported increased carbonyl (C=O) absorption at 1720 cm^−1^ and decreased aromatic C=C peaks around 1510 cm^−1^ in lignin after accelerated UV aging, reflecting oxidation and structural breakdown of phenolic groups [3,7]. Similarly, acrylic and polyurethane coatings show increased hydroxyl stretching (~3400 cm^−1^) and carbonyl formation, confirming chain scission and photo-oxidation [28,29]. Incorporating these spectral analyses complements the SEM observations, providing molecular-level evidence of coating and substrate degradation. For example, acrylic and polyurethane coatings undergo free radical generation, peroxyl radical conversion, chain scission, and crosslinking, collectively driving coating aging (Figure 4).

Beyond these general mechanisms, the photodegradation behavior of wood coatings is strongly dependent on the chemical structure of the film-forming resin. Different resin systems exhibit distinct degradation pathways and corresponding evaluation criteria. For clarity, the main degradation mechanisms, sensitive functional groups, and commonly used performance indicators for representative coating systems are summarized in Table 1.

Pigments and fillers exhibit distinct behaviors under photodegradation [29]. Organic pigments may decompose under light, causing fading or yellowing, while inorganic pigments (e.g., TiO_2_, ZnO) absorb or scatter UV light, protecting the resin from partial UV damage [30]. However, highly photocatalytic inorganic particles may accelerate oxidative degradation of surrounding polymers. UV-induced resin degradation combined with temperature-humidity cycles produces microcracks, increasing pathways for water and oxygen, while differential thermal expansion and moisture movement between the coating and substrate further cause blistering, peeling, or adhesion loss [31].

**Figure 5:**
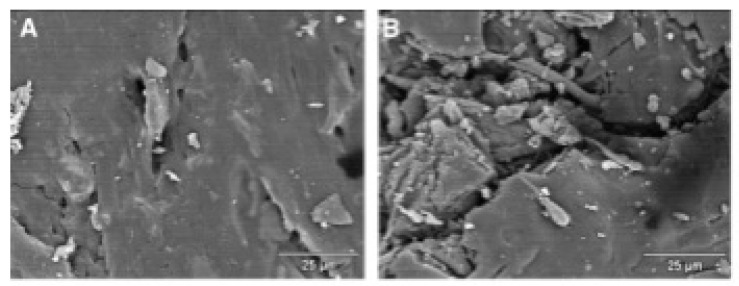
SEM image of the surface layer of wood-PP composites: (**A**) control non-weathered, (**B**) control after 1 year of weathering [32]. Scale bar: 25 μm.

For the wood substrate, UV radiation directly initiates lignin photodegradation, producing chromophoric compounds such as p-benzoquinone, causing yellowing or darkening. Water molecules, as mediators of photochemical reactions, generate additional free radicals under UV exposure, further accelerating photodegradation. This multi-factor UV–water–oxygen interaction constitutes the primary mechanism underlying wood coating aging. The SEM images in Figure 5 further confirm this mechanism: the surface is smooth before weathering, while obvious deterioration appears after one year of weathering [32].

## 4. Wood Coatings Anti-Photodegradation Strategies

### 4.1. UV Absorber Modification

#### 4.1.1. Mechanism of UV Absorbers

UV absorbers are among the earliest anti-aging additives applied in wood coatings. Their primary mechanism involves absorbing ultraviolet (UV) radiation and converting the energy into harmless heat, thereby reducing the amount of UV radiation reaching the wood substrate. This protective function relies on photophysical processes at the molecular level, such as the formation and breaking of intermolecular hydrogen bonds, intramolecular charge transfer (ICT), and energy transfer between excited molecules [33]. Studies indicate that incorporating UV absorbers into wood coatings significantly mitigates direct UV-induced damage to chemical bonds within the coating and delays aging caused by photo-oxidative reactions [34]. The effectiveness of UV absorbers strongly depends on their molecular structure; for instance, benzotriazole-based compounds efficiently convert light energy via the excited-state intramolecular proton transfer (ESIPT) mechanism, while benzophenone derivatives achieve similar protection through keto-enol tautomerization.

#### 4.1.2. Types and Characteristics

Common UV absorbers include benzotriazole, benzophenone, and other derivative compounds. Benzotriazole UV absorbers are widely used in wood coatings due to their strong UV absorption and excellent photostability. Their molecular structures contain multiple conjugated double bonds and aromatic rings, enabling effective absorption of UV radiation in the 280–400 nm range and converting it into harmless heat [35]. Experimental evidence shows that benzotriazole UV absorbers maintain high stability even under elevated temperatures, resisting decomposition or migration, making them particularly suitable for outdoor wood coatings [36].

Benzophenone-based UV absorbers are notable for their strong hydrogen bonding capability and high absorption coefficients. Their molecular structures contain carbonyl and hydroxyl groups that can form stable six-membered ring structures via intramolecular hydrogen bonds, enhancing photostability [37]. However, prolonged UV exposure may induce photodegradation of benzophenone derivatives, reducing their absorption efficiency. Therefore, in practical applications, they are often used in combination with other stabilizers to improve long-term durability.

Beyond these conventional UV absorbers, recent research has developed novel types such as metal–organic framework (MOF)-based nanoscopic absorbers and bio-based melanin nanoparticles. These advanced materials not only provide efficient UV shielding but also enable multifunctional integration through structural engineering, offering new strategies for improving the photostability of wood coatings [38].

#### 4.1.3. Application in Different Types of Wood Coatings

The performance and application of UV absorbers vary significantly across solvent-based, water-based, and UV-curable wood coatings. Their concentration and method of incorporation directly affect coating performance and weathering resistance. In solvent-based coatings, UV absorbers are typically added at low concentrations (0.1–1.0%) and dissolved directly in organic solvents, followed by high-speed stirring or ultrasonic dispersion to ensure uniform distribution. Benzotriazole absorbers are widely employed in such systems due to their excellent solubility and photostability; however, their migration tendency must be controlled through formulation optimization.

In water-based coatings, the incorporation of UV absorbers presents greater challenges due to the aqueous dispersion system. Hydrophilic UV absorbers or emulsification techniques are necessary to improve dispersibility. Moreover, higher loading levels (typically 1.0–3.0%) are required to compensate for potential concentration losses due to water evaporation.

In UV-curable coatings, the role of UV absorbers is even more critical. They not only protect the substrate from UV damage but also help to delay yellowing of the coating. Commonly, a combination of benzophenone and benzotriazole absorbers is used to achieve synergistic photostability. Careful attention must be paid to interactions between UV initiators and absorbers to avoid adverse effects on curing efficiency [39].

### 4.2. UV Stabilizer Modification

#### 4.2.1. Mechanism of UV Stabilizers

UV stabilizers are critical additives for enhancing the photostability of wood coatings. Their primary functions are to inhibit photo-oxidative reactions, scavenge free radicals, and quench excited-state molecules, thereby significantly improving the weathering resistance of the coating. Under UV irradiation, polymer chains within wood coatings can become excited, generating large amounts of reactive free radicals. These radicals attack the polymer chains, leading to cracking, chalking, and gloss loss, which seriously compromise the protective and decorative functions of the wood.

UV stabilizers operate through two main mechanisms: (1) Hindered amine light stabilizers (HALS) capture free radicals and convert them into stable nitroxyl radicals, interrupting the radical chain reaction; (2) Triazine-based stabilizers quench excited-state molecules, converting the excitation energy into harmless heat and thereby preventing further photo-oxidation [40].

The action mechanism of HALS is commonly described by the “Denisov cycle,” in which free radicals are continuously captured and active sites regenerated to achieve long-term photostabilization. Specifically, HALS molecules generate active nitroxyl radicals under UV exposure in the presence of oxygen. These radicals react with wood or polymer radicals to form intermediate species (e.g., aminoxy groups) and subsequently interact with peroxy radicals, reactivating HALS and generating harmless alcohols or ketones, thereby effectively delaying the photodegradation of both the coating and the wood substrate [41].

#### 4.2.2. Types and Characteristics

UV stabilizers can be broadly categorized into two classes: HALS and UV absorbers (UVA). HALS are further classified into three structural types: low-molecular-weight, polymer-grafted, and multifunctional HALS. Low-molecular-weight HALS dissolve easily but exhibit high migration; polymer-grafted HALS attach the hindered amine structure to the polymer backbone, reducing migration and enhancing durability; multifunctional HALS contain multiple hindered amine groups, allowing simultaneous scavenging of multiple radicals, resulting in higher efficiency. HALS can also be combined with UVAs to form synergistic photostabilization systems [40].

Common UVAs include benzotriazole, benzothiazole, and triazine derivatives. Benzotriazole UVAs have excellent UV absorption and high thermal stability, making them suitable for acrylic, polyurethane, and water-based coatings. Benzothiazole UVAs exhibit good optical transparency and are ideal for clear coatings and varnishes. Triazine UVAs are highly heat- and UV-resistant, particularly suited for outdoor high-durability coatings. By optimizing the combination of HALS and UVA, coatings can achieve synergistic protection, effectively blocking UV radiation and capturing free radicals, thereby prolonging the service life of wood products [41].

#### 4.2.3. Effect of UV Stabilizers

**Figure 6:**
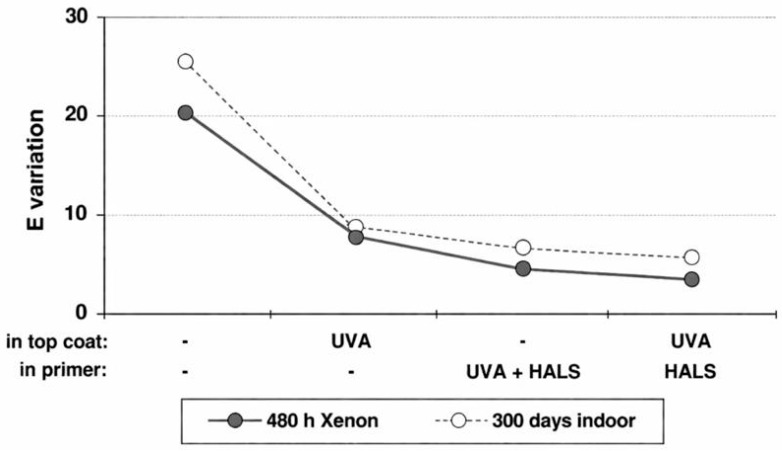
Effect of UVA-2 and HALS-1 on color change of pine [40].

Studies have demonstrated that incorporating HALS into wood coatings significantly delays yellowing and gloss loss induced by UV exposure. Pretreating coatings with HALS in waterborne or solvent-based systems, combined with UVAs, can effectively inhibit photodegradation of lignin on wood surfaces. This protection is particularly pronounced for light-colored woods such as pine and ash. Experiments show that when HALS-3 is added at 1–2% to a waterborne primer and coated with 2% UVA topcoat, a 500 h UVA-340 irradiation test resulted in a marked reduction in Δb* values, with color retention significantly improved compared to coatings without HALS (Figure 6). Even applying a single HALS primer layer provides noticeable protection to both coating and substrate, demonstrating the method’s practical feasibility and effectiveness.

### 4.3. Barrier and Shielding Material Modification

Barrier and shielding materials play a pivotal role in enhancing the photostability of wood coatings. Their primary mechanism is to form a dense physical or composite network within the coating, effectively blocking UV radiation from reaching the wood substrate and thereby slowing the photo-oxidative degradation of lignin and cellulose. Nanoparticles and natural polymer-based nanomaterials are widely used due to their high specific surface area and excellent optical and mechanical properties, making them highly effective for UV shielding [42].

In the realm of green nanomaterials, cellulose nanofibrils (CNFs), cellulose nanocrystals (CNCs), and lignin nanoparticles (LNPs) can form continuous dense films in waterborne coatings. These films improve the mechanical strength and water resistance of the wood surface while significantly enhancing UV-blocking performance. Accelerated heat–humidity aging tests have demonstrated that these natural nanomaterials further suppress photo-oxidative degradation and delay coating yellowing and surface chalking [43].

**Figure 7:**
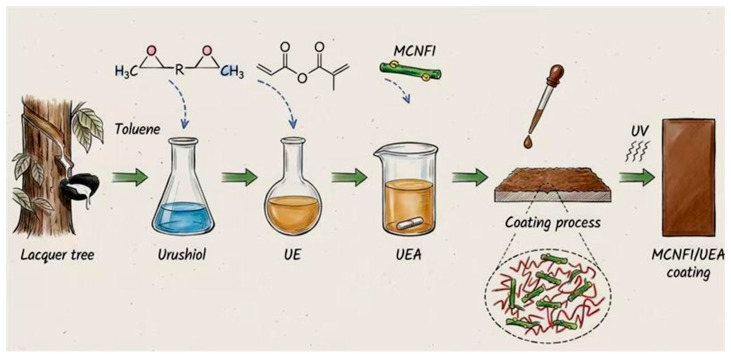
Schematic of MCNFI/UEA nanofiber composite coating structure for UV shielding and mechanical reinforcement of wood surfaces, adapted from [46].

Inorganic nanoparticles such as ZnO, TiO_2_, and CeO_2_ absorb or scatter UV light, effectively blocking UVA and UVB penetration, thus extending the service life of both the coating and the wood substrate [44,45]. Surface modification or composite strategies allow these nanoparticles to be evenly dispersed within the coating, minimizing aggregation while improving adhesion and hydrophobicity. This achieves a balance between high transparency and high UV-blocking performance [46]. As illustrated in Figure 7, MCNFI/UEA nanofiber composite coatings form a three-dimensional crosslinked network, synergistically combining nanoparticles and resin to enhance UV shielding, mechanical stability, and durability on the wood surface.

Moreover, magnetic nanoparticles and composite nanocoatings have been developed to block UV light and heat transfer. For example, Fe_3_O_4_@urushiol–iron polymer composite nanoparticles form an efficient barrier layer on the coating surface, improving both UV stability and long-term durability [47]. In summary, barrier and shielding materials slow the photodegradation of wood coatings through physical blocking, UV absorption, scattering, and interfacial synergy. Future research should focus on optimizing nanoparticle dispersion, particle size distribution, and composite strategies to achieve the combined benefits of high transparency and strong UV shielding, providing a theoretical basis and technical guidance for the development of high-performance wood coatings [46].

### 4.4. Antioxidant Modification

Antioxidants play a crucial role in wood coatings by capturing free radicals, decomposing peroxides, and interrupting photo-oxidative chain reactions, thereby effectively slowing the photochemical degradation of both the polymer matrix and the wood substrate [10,48]. During photo-oxidation, UV radiation excites resin molecules to form free radicals, which rapidly attack polymer chains and lignin, leading to resin degradation, wood yellowing, and coating chalking. Antioxidants can supply hydrogen atoms or form stable complexes with free radicals, significantly suppressing these chain reactions and extending the service life of both the coating and the wood [49].

Antioxidants are generally classified into primary antioxidants and secondary antioxidants. Primary antioxidants, such as hindered phenols, directly donate hydrogen atoms to terminate free radical chain reactions, whereas secondary antioxidants, such as phosphite esters, decompose peroxides to form stable products, thereby delaying coating oxidation [10,49]. Experiments indicate that primary antioxidants generally outperform secondary antioxidants in improving photostability, and when combined with UV absorbers or HALS, significant synergistic effects can be achieved [50].

**Figure 8:**
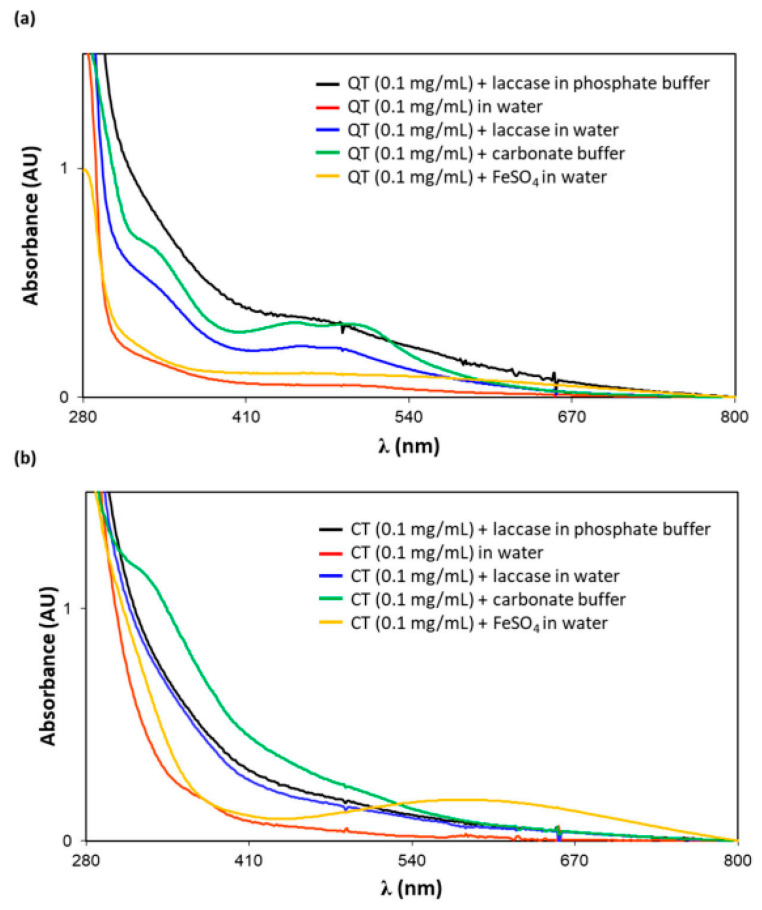
UV–vis spectra of dip coating solutions after 2 h immersion. (**a**) Condensed tannin (QT); (**b**) Hydrolyzable tannin (CT) [52].

In recent years, natural bio-based antioxidants such as lignin, tannins, and polyphenols have attracted considerable attention. These natural antioxidants, due to their aromatic hydroxyl structures, can effectively scavenge free radicals while providing inherent UV-absorbing properties, enabling multifunctional photoprotection [51,52,53]. For instance, Moccia et al. (2020) compared condensed tannins (QT) with hydrolyzable tannins (CT) in terms of coating formation and antioxidant performance [52]. Results showed that coatings formed from condensed tannins exhibited stronger antioxidant capacity, forming more stable, leach-resistant, and functional coatings (Figure 8). These findings provide a direct basis for selecting and designing natural antioxidants in wood coatings. Additionally, composite antioxidant systems, such as lignin-nanofiber or chitosan-polydopamine composites, have been shown to form highly protective surface films, further enhancing photostability [54].

In summary, antioxidants protect resin and lignin structures at the molecular level, significantly improving the photostability of coatings and wood. Rational selection of antioxidant type, dosage, and combination strategy—particularly the synergistic use of natural and synthetic antioxidants—is key to achieving high-performance, photostable wood coatings.

It should be noted that antioxidants used in wood coatings can serve different functions depending on the stage of application. During lacquer synthesis or storage, antioxidants (typically hindered phenols or amine-based stabilizers) are primarily employed to inhibit premature oxidation and prevent gelation, thereby ensuring formulation stability. In contrast, antioxidants incorporated into the applied coating are intended to suppress photo-oxidative degradation during service life by scavenging free radicals or decomposing peroxides.

These two applications require different selection criteria, as not all antioxidants suitable for storage stability are appropriate for long-term photostabilization. In particular, certain antioxidants are prone to discoloration under UV exposure. Hindered phenolic antioxidants may form quinone-type oxidation products, leading to yellowing of the coating. Similarly, aromatic amine antioxidants can undergo photo-oxidation to generate colored species, contributing to darkening or staining. In contrast, phosphite antioxidants generally exhibit lower discoloration tendency but are less effective as primary radical scavengers. Therefore, careful selection and combination of antioxidants are necessary to balance processing stability and long-term optical performance.

### 4.5. Comparison of Photostabilization Strategies

The main strategies for enhancing the photostability of wood coatings include UV absorber modification, UV stabilizer modification, barrier and shielding material modification, and antioxidant modification. Each strategy differs significantly in mechanism, material applicability, durability, and overall performance. Rational combination of these strategies can achieve synergistic effects. Table 2 provides a comparative analysis of mechanisms, advantages, disadvantages, and typical applications.

To further illustrate the effectiveness of different strategies, Table 3 summarizes quantitative improvements in key properties such as color stability, UV shielding, and durability reported in the literature.

### 4.6. State-of-the-Art Developments in Wood Coating Photostabilization

Recent advances in wood coating photostabilization have shifted from single-function additives toward multifunctional and integrated systems, emphasizing synergy between UV shielding, radical scavenging, and structural reinforcement.

One prominent trend is the development of organic–inorganic hybrid nanocomposites, such as cellulose nanocrystals (CNCs) combined with metal oxides (e.g., CeO_2_, ZnO), which significantly enhance UV shielding while maintaining coating transparency and mechanical performance [11,38]. Surface-modified nanoparticles and core–shell structures further improve dispersion and interfacial compatibility, addressing traditional limitations of nanoparticle aggregation.

Another emerging direction involves bio-based photostabilizers, including lignin nanoparticles, tannins, and polydopamine-derived systems. These materials provide inherent UV absorption and antioxidant properties due to their aromatic and phenolic structures, enabling sustainable and multifunctional coating design [51,52,53]. However, challenges remain in terms of long-term stability and compatibility within polymer matrices.

In addition, synergistic stabilization systems, combining UV absorbers, HALS, and antioxidants, have demonstrated significantly improved durability compared to single-additive systems. Recent studies report that such hybrid strategies can enhance photostability by more than 50% under accelerated aging conditions [55,56].

Beyond mechanistic descriptions, recent studies increasingly emphasize the direct correlation between photodegradation mechanisms and macroscopic performance. For instance, polymer chain scission and photo-oxidation lead to a reduction in molecular weight and crosslink density, resulting in decreased mechanical properties, such as lower tensile strength and increased brittleness. At the same time, the formation of chromophoric groups (e.g., carbonyl and quinone structures) during lignin degradation contributes to optical deterioration, including color change (ΔE, Δb*) and gloss loss.

From a durability perspective, microcrack formation and interfacial degradation induced by UV–moisture coupling significantly accelerate the ingress of water and oxygen, thereby reducing long-term weathering resistance. Conversely, the incorporation of UV absorbers, HALS, and nanomaterials has been shown to mitigate these effects, leading to measurable improvements in color stability, gloss retention, mechanical integrity, and service life.

These findings highlight that effective photostabilization strategies should be evaluated not only at the molecular level but also in terms of their impact on macroscopic performance, establishing a critical link between mechanism and practical application.

Furthermore, advanced functional coatings, including superhydrophobic surfaces, UV-responsive materials, and smart coatings with adaptive protection mechanisms, are gaining increasing attention. These systems aim to simultaneously improve moisture resistance, UV shielding, and environmental adaptability, representing a promising direction for next-generation wood coatings.

Overall, the state of the art highlights a clear transition toward multi-scale, multifunctional, and sustainable photostabilization strategies, while challenges related to durability, compatibility, and large-scale application remain to be addressed.

**Figure 9:**
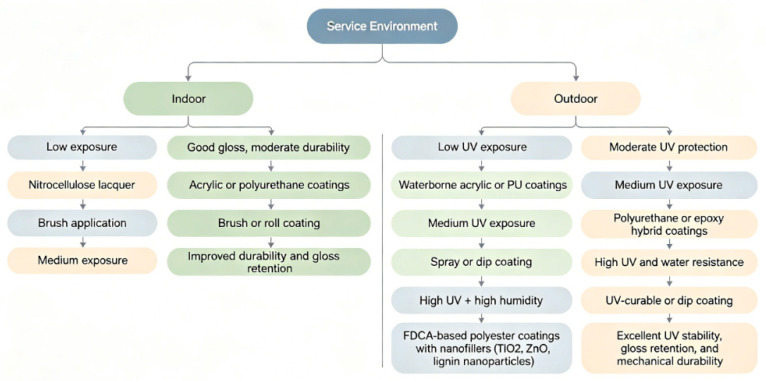
Decision flowchart for selecting wood protective coatings based on service conditions.

To facilitate practical application, a decision flowchart has been developed to guide the selection of appropriate wood protective coatings and corresponding application methods based on specific service conditions. Factors considered include indoor versus outdoor use, UV exposure intensity, moisture level, and required performance metrics such as gloss retention and color stability. This schematic (Figure 9) provides a concise visual reference for selecting suitable materials and processing techniques.

Recent advances in wood coatings emphasize sustainable and multifunctional polymer systems. Bio-based monomers, such as 2,5-furandicarboxylic acid (FDCA), have been incorporated into polyester coatings to improve UV resistance, mechanical performance, and thermal stability compared with conventional petrochemical-based polyesters. Hybrid formulations combining acrylic, polyurethane, and epoxy resins with nanofillers, including TiO_2_, ZnO, or lignin nanoparticles, provide synergistic improvements in photostability, water resistance, and gloss retention [11,23,46]. Waterborne and UV-curable coatings continue to gain attention due to lower VOC emissions, faster curing, and tunable optical properties [39,40]. Collectively, these modern coating strategies illustrate a trend toward environmentally friendly, high-performance, and multifunctional wood protection systems.

## 5. Challenges and Future Directions of Photostabilization in Wood Coatings

### 5.1. Challenges

Despite significant progress in enhancing the photostability of wood coatings, several challenges remain in practical applications. First, the long-term UV resistance of coatings is limited by the synergistic effects of ultraviolet radiation and environmental factors. UV exposure, temperature–humidity cycling, and atmospheric pollutants collectively accelerate the photo-oxidative degradation of both the coating and wood substrate, reducing the effectiveness of protective systems during prolonged outdoor exposure [14]. Second, the dispersion, stability, and compatibility of nanofillers and functional additives within coating formulations are still limited, which may lead to uneven protection, localized degradation, or compromised mechanical properties. Additionally, although natural antioxidants and bio-based materials offer environmental benefits and multifunctionality, their complex structures and photo-labile active components often hinder long-term stability within the coating [51,52]. Overall, achieving a balanced combination of high transparency, strong UV-blocking capability, and long-term durability remains a core challenge for photostable wood coatings.

### 5.2. Future Directions

To address these challenges, future research on photostable wood coatings should focus on several key directions. First, the development of efficient composite protective systems that synergistically combine UV absorbers, hindered amine light stabilizers (HALS), and natural antioxidants can provide multi-level protection through simultaneous free radical scavenging and UV shielding, while optimizing the coating microstructure to enhance both mechanical and optical performance [55,56]. Second, the exploration of smart nanomaterials and green bio-based additives, such as cellulose nanofibers, lignin nanoparticles, and chitosan–ZnO composites, can improve light stability, water resistance, and environmental adaptability through multi-scale structural design [57,58]. Third, integrating photocatalytic inhibition, thermal-response regulation, and multifunctional composite strategies may enable coatings to exhibit adaptive protection under varying environmental conditions [59,60]. Fourth, strengthening standardized evaluation protocols and conducting long-term outdoor experiments are essential for systematically quantifying photodegradation and providing data-driven support for high-performance coating optimization [61,62]. In summary, the future development trend of wood coatings lies in simultaneously achieving material sustainability, multifunctionality, and intelligent mechanisms, paving the way for coatings that combine superior photostability, environmental friendliness, and high performance.

## 6. Conclusions

This review systematically summarized the photodegradation characteristics, influencing factors, and anti-aging strategies of wood coatings. Photodegradation in wood coatings involves polymer matrix chain scission, chemical structure reorganization, and photo-oxidation of the wood substrate, leading to coating chalking, cracking, loss of gloss, and color changes, which severely compromise the mechanical performance and service life of wood. Current strategies to mitigate photodegradation include the use of UV absorbers to convert ultraviolet radiation into harmless heat, HALS-type UV stabilizers to capture free radicals and quench excited-state molecules, barrier materials that form physical nano-scale shields while enhancing mechanical properties and water resistance, and antioxidants that remove free radicals or decompose peroxides at the molecular level. These approaches can be applied individually or in combination to achieve synergistic protection.

However, long-term outdoor exposure remains challenging, as the synergistic effects of UV radiation, temperature–humidity cycling, and environmental pollutants still limit coating performance. The activity of natural antioxidants tends to decrease over time, the dispersion and compatibility of nanoparticles require further optimization, and achieving a balance between high transparency and strong UV-blocking ability remains difficult.

Future research should focus on designing multifunctional composite coatings that integrate natural bio-based materials and nanotechnology to enhance photostability, mechanical performance, and water resistance. The development of smart responsive materials, adaptive protection mechanisms, and standardized long-term evaluation protocols will provide scientific guidance and technical support for high-performance wood coatings, while also promoting the high-value utilization and sustainable development of wood resources.

## Figures and Tables

**Table 1:** Degradation mechanisms and evaluation criteria for representative film-forming resins in wood coatings.

Resin Type	Main Degradation Mechanism	Sensitive Groups	Key Evaluation Criteria
Alkyd	Photo-oxidation, chain scission	C=C, ester	Carbonyl index, Δb, gloss
Polyurethane	Urethane cleavage, oxidation	-NH-COO-	ΔE, tensile strength
Epoxy	Ether bond scission	C-O-C	Crosslink density, FTIR
Nitrocellulose	Nitrate photolysis	-ONO_2_	Mass loss, gloss

**Table 2:** Comparison of different photostabilization strategies for wood coatings, including their mechanisms, advantages, disadvantages, applicable coating systems, and representative references.

Strategy	Mechanism	Advantages	Disadvantages	Applicable Systems	Representative References
UV Absorbers	Absorb UV light and convert it into heat, reducing UV energy penetration	High UV absorption efficiency; suitable for transparent coatings; compatible with other additives	May degrade under prolonged UV exposure; partial migration	Solvent-based, waterborne, UV-curable coatings	[40,47]
UV Stabilizers (HALS)	Capture free radicals and quench excited-state molecules, interrupting photo-oxidative chain reactions	Long-term effectiveness; synergistic with UV absorbers; suitable for high-durability coatings	High concentrations may affect transparency; limited compatibility with acidic systems	Acrylic, polyurethane, waterborne coatings	[40,41]
Barrier & Shielding Materials	Form dense coatings with nanoparticles or nanofibers that scatter or absorb UV	Improve mechanical strength and water resistance; enhance protection of natural wood	Nanoparticle aggregation may reduce transparency; higher production cost	Waterborne coatings, composite coatings, outdoor wood	[43,46]
Antioxidants	Capture free radicals or decompose peroxides, interrupting photo-oxidative chain reactions	Improve stability of resin and lignin; synergistic with UV absorbers and HALS	Sensitive to combined thermal and UV stress; natural antioxidants may have dispersion issues	Solvent-based, waterborne, bio-based coatings	[10,52]

**Table 3:** Quantitative performance improvements of wood coatings with different photostabilization strategies reported in the literature.

Strategy	Additive/System	Key Property	Before Modification	After Modification	Improvement	Reference
UV Absorber	Benzotriazole (UVA)	ΔE (color change) after UV exposure	~12–15	~5–7	↓ ~50–60%	[40]
HALS + UVA	HALS-3 + UVA-2	Δb* (yellowing) after 500 h UVA-340	High yellowing	Significantly reduced	↓ ~40–60%	[40]
Nanoparticles	ZnO/TiO_2_	UV transmittance (UVA)	~60–70%	<20%	↓ ~70%	[44,45]
Lignin nanoparticles	LNPs (~1%)	UVA transmittance	~50%	<10%	↓ ~80%	[23]
Cellulose/CeO_2_ composite	CNC + CeO_2_	UV shielding efficiency	Moderate	High	↑ significant	[11,38]
Antioxidants	Condensed tannins	Radical scavenging/stability	Moderate	Enhanced stability	↑	[52]
Hybrid systems	HALS + nanoparticles	Service life/durability	Baseline	>50% increase	↑ >50%	[20,55]

## Data Availability

The original contributions presented in this study are included in the article. Further inquiries can be directed to the corresponding author.
